# Novel Titanium Nanospike Structure Using Low-Energy Helium Ion Bombardment for the Transgingival Part of a Dental Implant

**DOI:** 10.3390/nano12071065

**Published:** 2022-03-24

**Authors:** Khaled Mukaddam, Monika Astasov-Frauenhoffer, Elizaveta Fasler-Kan, Laurent Marot, Marcin Kisiel, Roland Steiner, Fabien Sanchez, Ernst Meyer, Joachim Köser, Michael M. Bornstein, Sebastian Kühl

**Affiliations:** 1 Department of Oral Surgery, University Center for Dental Medicine Basel (UZB), University of Basel, Mattenstrasse 40, 4058 Basel, Switzerland; sebastian.kuehl@unibas.ch; 2 Department Research, University Center for Dental Medicine Basel (UZB), University of Basel, Mattenstrasse 40, 4058 Basel, Switzerland; m.astasov-frauenhoffer@unibas.ch; 3 Department of Biomedicine, University Hospital Basel, University of Basel, Hebelstrasse 20, 4031 Basel, Switzerland; e.fasler@unibas.ch; 4 Department of Pediatric Surgery, Children’s Hospital, Inselspital Bern, University of Bern and Department of Biomedical Research, University of Bern, Freiburgstrasse 15, 3010 Bern, Switzerland; 5 Department of Physics, University of Basel, Klingelbergstraße 82, 4056 Basel, Switzerland; laurent.marot@unibas.ch (L.M.); marcin.kisiel@unibas.ch (M.K.); roland.steiner@unibas.ch (R.S.); fabien.sanchez@unibas.ch (F.S.); ernst.meyer@unibas.ch (E.M.); 6 Institut für Chemie und Bioanalytik, Hochschule für Life Sciences, Hofackerstrasse 30, 4132 Muttenz, Switzerland; joachim.koeser@fhnw.ch; 7 Department of Oral Health & Medicine, University Center for Dental Medicine Basel (UZB), University of Basel, Mattenstrasse 40, 4058 Basel, Switzerland; michael.bornstein@unibas.ch

**Keywords:** antibacterial, titanium, nanospike, surface, gingival fibroblast

## Abstract

Aim(s): The aim of the study was to fabricate a nanospike surface on a titanium alloy surface using a newly established method of low-energy helium ion bombardment. Various methods to achieve nanospike formation on titanium have been introduced recently, and their antibacterial properties have been mainly investigated with respect to *Escherichia coli* and *Staphylococcus aureus*. Oral pathogens such as *Porphyromonas gingivalis* play an important role in the development of peri-implantitis. For that reason, the antibacterial properties of the novel, nanostructured titanium surface against *P. gingivalis* were assessed, and a possible effect on the viability of gingival fibroblasts was evaluated. Materials and Methods: Helium sputtering was employed for developing titanium surfaces with nanospikes of 500 nm (ND) in height; commercially available smooth-machined (MD) and sandblasted and acid-etched titanium disks (SLA) were used as controls. Surface structure characterization was performed through scanning electron microscopy (SEM) and atomic force microscopy (AFM). Following incubation with *P. gingivalis*, antibacterial properties were determined via conventional culturing and SEM. Additionally, the viability of human gingival fibroblasts (HGFs) was tested through MTT assay, and cell morphology was assessed through SEM. Results: SEM images confirmed the successful establishment of a nanospike surface with required heights, albeit with heterogeneity. AFM images of the 500 nm nanospike surface revealed that the roughness is dominated by large-scale hills and valleys. For frame sizes of 5 × 5 μm and smaller, the average roughness is dominated by the height of the titanium spikes. ND successfully induces dysmorphisms within *P. gingivalis* cultures following the incubation period, while conventional culturing reveals a 17% and 20% reduction for ND compared to MD and SLA, respectively. Moreover, the nanospike surfaces did not affect the viability of human growth fibroblasts despite their sharp surface. Conclusion(s): This study successfully developed a novel titanium-nanospike-based structuration technique for titanium surfaces. In addition, the nanospikes did not hinder gingival fibroblast viability. Enhanced antimicrobial effects for such a novel nanospike-based resurfacing technique can be achieved through further optimizations for nanospike spacing and height parameters.

## 1. Introduction

The development and utilization of osseointegrated dental implants is one of the main routine dental surgery procedures applied in dental patient care on a global scale, providing major benefits in patients with tooth loss caused by a spectrum of conditions. However, as with any other surgical procedure, such implants do carry with them a risk of peri- and post-procedural complications. One of these complications is peri-implantitis, which typically manifests itself within the patient via the development of a microbial biofilm over the dental implant surface—especially on the transgingival part of the implant [1]. Peri-implantitis can lead to acute infections and induce severe inflammatory outcomes that ultimately damage peri-implant tissue, in a similar manner to permanent periodontal disease. However, unlike periodontal disease, the pathogenesis of peri-implantitis can be more widespread, leading to more rapid tissue devastation in such cases [2].

Typically, peri-implantitis, due to microbial biofilm development, is affected by multiple factors, including the biofilm-colonizing causative microbial agent(s), as well as the dental implant material with its unique surface topography. In addition, the fact that the oropharynx provides a level growth field for a myriad of micro-organisms due to the variety of food consumed by the individual, coupled with the fact that oral mucosa is continuously shed, leads to the prerequisite that biofilm-developing microbial colonies form an ecosystem within the oral cavity [3]. However, biofilms that develop on the hardened surfaces of native teeth or dental implants, which are not able to shed outer cellular layers, allow for the consequent accumulation of biofilms, inducing dental conditions that include periodontitis and peri-implantitis [3]. Furthermore, microbial biofilm development is facilitated on rough surfaces, consequently proving that material selection is pivotal for dental implant survival [3,4].

Although peri-implantitis-inducing microbial biofilms can consist of diverse microbial species, retrospective studies have revealed that the vast majority of dental implant peri-implantitis cases involve overwhelming colonization by *Porphyromonas gingivalis*, followed by *Prevotella intermedia* and *Prevotella nigrescens* [5,6]. *P. gingivalis* is a Gram-negative anaerobic bacterium with previously established notoriety as a pathobiont—being present in the oral biome with the ability to proliferate rapidly within periodontal regions of the oral cavity [7]. In addition, *P. gingivalis* is also capable of thwarting the innate immune system of the host, together with exacerbating inflammation, due to a spectrum of virulence-inducing components such as gingipains and fimbriae, together with the presence of lipopolysaccharides and capsule formation [8]. Previous in vivo studies on peri-implantitis using murine models highlighted the issue that *P. gingivalis*-driven peri-implantitis leads to exacerbations in bone loss within implant regions, rather than native tooth regions [9].

With the knowledge of the etiology and pathogenesis of *P. gingivalis*-driven peri-implantitis, the design and development of the appropriate materials for use in dental implants is crucial—especially since the unique topography of such materials can directly affect the risk of *P. gingivalis*-colonized biofilm accumulation [10]. Ultimately, the long-term success rate for osseointegrated dental implant surgical procedures depends on the ability of the dental implant surfaces to fend off—as well as possible—any attempted *P. gingivalis*-based biofilm development on the transgingival part of the dental implant or abutment surfaces.

Regarding the development of antibacterial dental implant surfaces, lessons can be learned through the study of defense mechanisms adopted by other organisms [11,12]. All such naturally occurring surfaces have in common the ability to counteract microbial threats and, consequently, are a source of inspiration for mimicking such topographies onto dental implants (among other potential bio-utilized prosthetics) for the purpose of conferring antibacterial effects and preventing consequent biofilm accumulation [12].

Previous studies have developed multiple variations on such nanopillar/spike surface structures through employing a spectrum of materials, including silicon, hydroxyapatite, diamond, gold, and titanium [13,14,15,16,17,18]. Thus, such a technology was able to be successfully implemented as an antibacterial prophylactic measure on selected orthopedic implants [19,20].

The study conducted by Bhadra et al. in 2015 adopted the model employed by dragonfly wings, with nanopatterned surfaces, for the development of a bespoke titanium nanowire topography on dental implants. Apart from having effective antibacterial capacity, such bespoke nanowire surfaces on the dental implant enabled enhanced proliferative growth and development of primary human fibroblasts over the course of 10 days [13].

Another highly important study was carried out in 2018 by Hazell et al., wherein black silicon structures with nanoneedle-covered surfaces were analyzed for antibacterial properties. The study concluded that black silicon and black diamond surfaces (the latter being developed artificially from black silicon) offer a less accommodating environment for bacterial culture attachment and growth [15]. The investigation performed by Saraeva et al. in 2020 focused on the development of nanospikes through laser ablation of a 500 nm thick gold film, followed by analyzing the antibacterial properties—utilizing live/dead staining techniques—of this nanostructured film pre- and post-ablation [18].This study highlighted how nanospike-bearing gold film can successfully exhibit antibacterial properties. This is due to the increased red propidium iodide staining of dead bacteria, which is a result of punctured bacterial cell membranes [18]. The recent study carried out earlier this year by Elliott et al. adopted alkaline hydrothermal treatment for engineering a titanium alloy surface, for the generation of nanospikes ranging between 250–350 nm (following a 4 h treatment) and 100–1250 nm (following an 8 h treatment). This investigation additionally demonstrated that the 8 h alkaline, hydrothermally treated nanospike titanium alloy surface managed to kill close to 40% of static bacterial cultures following a one-hour-long incubation period [21].

The antibacterial effects provided by nanopillar/spike resurfacing are mediated through mechanical piercing of bacterial cell walls and/or cell membranes, together with impeding appropriate adhesive functionalities typically adopted by bacterial pathogens [22]. The nanopillar/spike characteristics should have the capacity to penetrate both Gram-negative and Gram-positive bacterial species, as the discrepancy in bacterial cell wall thickness can have a major influence on the nanopillar/spike design’s antibacterial efficacy. The study carried out by Ivanova et al. utilized silicon nanopillar arrays (35 nm diameter/90 nm periodicity) with three heights (220, 360, and 420 nm), which were developed using deep UV immersion lithography, with the 360 nm nanopillar found to have antibacterial properties against both Gram-negative *Pseudomonas aeruginosa* and Gram-positive *Staphylococcus aureus* bacterial colonization of the nanopillar surface [23]. The study carried out by Jenkins et al. adopted a thermal oxidation methodology for developing nanopillars over grade 5 titanium alloy (Ti-6A1-4V), followed by a five-minute high-temperature treatment (850 °C), leading to nanopillar formation [24]. Such nanopillars were found to be effective in thwarting bacterial colonization by *Escherichia coli* and *Klebsiella pneumoniae* (both Gram-negative bacterial species), as well as by—although to a reduced effect—Gram-positive *Staphylococcus aureus* bacterial populations [24].

In order to be able to use such a technology adequately for the transgingival part of dental implants, the surface must support soft tissue integration [25]. However, no consensus has been reached on the ideal nanostructural design for conferring antibacterial properties to the transgingival part of dental implants, thus creating a vacuum for such a research niche.

In essence, the aim of this study was to fabricate nanospikes with 500 nm spike dimensions on titanium alloy surfaces using a newly established method of low-energy helium ion bombardment. Various methods to achieve nanospike formation on titanium have been introduced recently, and their antibacterial properties have been mainly investigated towards *Escherichia coli* and *Staphylococcus aureus* [26]. Oral pathogens such as *P. gingivalis* play an important role in the development of peri-implantitis, which can lead to implant loss. As a result, the antibacterial properties of the novel, nanostructured titanium surface against *P. gingivalis* were assessed, and a possible effect on the viability of gingival fibroblasts was evaluated. It is also important to highlight that, to the best of the authors’ knowledge, no previous studies have been performed to develop nanopillar/nanospike surfaces for dental implants that can exert antibacterial properties against the major causative agents for peri-implantitis, such as *P. gingivalis*—although such titanium-based nanospikes have been found to confer antibacterial properties against other bacterial populations, as described above. This renders such research efforts highly attractive for possibly developing novel nanospike-coated dental implants that could have long-lasting efficacy against the risk of peri-implantitis within recipient patients in future clinical settings.

## 2. Material and Methods

### 2.1. Preparation of Specimen and Material Characterization

Heated titanium samples were exposed to helium (He) plasma using an unbalanced magnetron sputtering source; by controlling the temperature (350 °C), the negative voltage on the sample (−120 V), and the time of exposure, the surface developed a nanostructured pattern (data in submission process). The spikes’ formation was reported for the first time in 2014 by Kajita et al., who claimed that the process is related to physical mechanisms that cause the various morphological changes, including the following: the penetration and diffusion of irradiating helium atoms, helium bubble formation near the surface, physical sputtering, and redeposition of radicals. In particular, it seems that physical sputtering brings about significant difference from the morphological changes in tungsten, where the effect of the physical sputtering is negligible when the incident ion energy is less than 100 eV [27].

Three bespoke 15 mm diameter titanium discs with a 1.5 mm thickness were employed in this study, with the custom-made study group consisting of nanostructured discs with a 500 nm long nanospiked surface (ND). In the present study, the control groups consisted of commercially available smooth-machined titanium discs (MD) with a polished surface obtained through a grinding process, which also corresponded to the clinically standardized polished and smooth regions typically used for tissue-level dental implants and SLA^®^ titanium discs (SLA), which are sandblasted and acid-etched (Institute Straumann AG, Basel, Switzerland). The SLA surface, which corresponds to the implant’s osseointegrated part, was used as an additional control group. All tested control groups were manufactured by Institute Straumann AG, Basel, Switzerland.

#### 2.1.1. Scanning Electron Microscopy (SEM)

The specimens’ surfaces were gold-sputtered and visualized with a scanning electron microscope (SEM, field emission at 5 kV) (Hitachi, Tokyo, Japan).

#### 2.1.2. Atomic Force Microscopy (AFM

AFM experiments were performed in a dry environment, under a nitrogen gas atmosphere and at room temperature. Three samples (ND, MD, and SLA) were examined by means of atomic force microscopy (AFM) in intermediate-contact and full-contact modes. The topographic images and roughness were obtained in intermediate-contact mode AFM with a Nanosensors PPP-NC cantilever (Nanosurf, Liestal, Switzerland). The sensor stiffness and frequency were equal to k = 28 N/m and f = 160 kHz, respectively. Six different AFM images of every surface were taken, successively reducing the image size from 20 μm^2^ down to 0.3 μm^2^. The adhesion force and adhesion energy measurements were performed in contact mode AFM with a PPP-CONT cantilever, with sensor stiffness and frequency equal to k = 0.1 N/m and f = 11 kHz, respectively. To obtain adhesion values, 30 force–distance curves on every surface were acquired.

### 2.2. Adhesion of P. gingivalis on Specimens

A 10 µL sample of *Porphyromonas gingivalis* (ATCC 33277) stock solution was inoculated to 10 mL of thioglycolate (BBL^TM^, Becton Dickinson, Allschwil, Switzerland) enriched with 0.5 mg/L menadione (Merck, Buchs, Switzerland) and 5 mg/L hemin Merck, Buchs, Switzerland), and the culture was incubated for 96 h in anaerobic conditions at 37 °C. Thereafter, the bacteria were harvested in a stationary growth phase by centrifugation (8500 rpm, 5 min, RT), resuspended in 10 mL of simulated body fluid [28] enriched with 0.2% glucose, and allowed to adhere to the three different material groups—ND, MD, and SLA—for 6 h, at 37 °C, in static anaerobic conditions.

Thereafter, the discs were gently dipped in 0.9% NaCl (Merck, Buchs, Switzerland), with bacterial cultures being either harvested and cultivated by conventional culturing on Columbia blood agar (BBL^®^, BD Becton Dickinson™, Allschwil, Switzerland), or fixed in 2% glutaraldehyde (Merck, Buchs, Switzerland), dehydrated in stepwise increasing concentrations of ethanol, critical-point dried, and coated with 10 nm of gold for scanning electron microscopy (SEM) [25]. Consequently, inoculated plates were incubated for seven days at 37 °C in anaerobic conditions, prior to assessment of colony-forming units for each individual titanium disc, as previously described [29].

The reduction in adhesion was calculated using the following formula:

reduction% = 100 × ((CFU_M or SLA_ − CFU_ND_)/CFU_M or SLA_).

### 2.3. Specimen Interaction with Human Gingival Fibroblasts

Human gingival fibroblast (HGF) cells (CRL-2014) were purchased from the ATCC (Manassas, VA, USA), accompanied by identification test certificates. The cells were grown in Dulbecco’s minimal essential growth medium (DMEM), according to standardized tissue culturing protocols, using 10% fetal bovine serum (FBS) and 1% penicillin–streptomycin solution at 37 °C, 5% CO_2_, and 100% humidity. Across all experiments conducted in this study, HGF cultures were employed for passages 3–8. In addition, FBS and DMEM (BioConcept™, Allschwil, Switzerland) were used together with typical cell culturing reagents (i.e., trypsin–EDTA, penicillin–streptomycin solution, stable glutamine) (Biowest™, Nuaillé, France). All cell culture experiments were performed using TPP™ (Trasadingen, Switzerland) plasticware. Subsequent experimental protocols were as follows:

#### 2.3.1. MTT Assay

To evaluate the effects of the various surfaces (ND, MD, and SLA) on gingival fibroblasts, an MTT cell viability assay was performed. In total, 30,000 HGF cells were cultured on various discs within 24-well plates for 72 h, followed by the addition of thiazolyl blue tetrazolium bromide (MTT) at a concentration 0.1 mg/mL. HGF cells were consequently incubated for a further 4 h, and the reaction was finally stopped by adding 125 μL of dimethyl sulfoxide (DMSO). MTT and DMSO were purchased from Merck™ (Buchs, Switzerland). All supernatants were harvested, and the optical density was measured at 590 nm, as previously described [29].

#### 2.3.2. Cell Morphology by Scanning Electron Microscopy (SEM)

First, 4 × 10^4^ HGF-1 cells were seeded on the specimen surface (ND, MD, and SLA). After 24 h of culture, cells were washed twice with phosphate buffered saline (PBS) and fixed with 2% glutaraldehyde in PBS for 30 min. Glutaraldehyde was removed and free aldehyde groups were quenched by adding 1 mL of 0.1 M glycine in PBS. Cells were washed twice with PBS and subsequently fixed with 2% osmium tetroxide in 0.1 M cacodylate buffer and incubated for 30 min. Cells were washed twice with cacodylate buffer. Dehydration was performed with graded ethanol (twice each with 50, 70, 90, and 100% ethanol for 2 min. Samples were critical-point dried with CO_2_ (Critical Point Dryer, CPD 030, BAL-TEC) and sputtered (SCD 050, Sputter Coater, BAL-TEC AG, Balzers, Liechtenstein) with approximately 50 nm Au–Pd to make the cells electroconductive. Cell morphology was visualized with SEM (ESEM XL30, Philips, Eindhoven, the Netherlands), which was performed once, with duplicate probes (*n* = 2).

Unless otherwise stated, all reagents, chemicals, culture media, serum and PBS were purchased from Sigma–Aldrich Inc. (St. Louis, MO, USA).

## 3. Statistical Analysis

Data were collected on an Excel sheet (Microsoft Corporation, Richmond, CA, USA) for descriptive analysis. The normality of the results was tested using the Shapiro–Wilk test, and Student’s *t*-test was applied (IBM^®^, SPSS^®^ Statistics software Version 26.0 (IBM Corp., Armonk, NY, USA)) to assess statistically significant differences between the adhesion and viability of *P. gingivalis* and the viability of gingival fibroblasts on ND, SLA, and MD discs in the experiments conducted. The level of significance was set to *p* < 0.05.

## 4. Results

### 4.1. Specimen Characterization

#### 4.1.1. SEM

**Figure 1:**
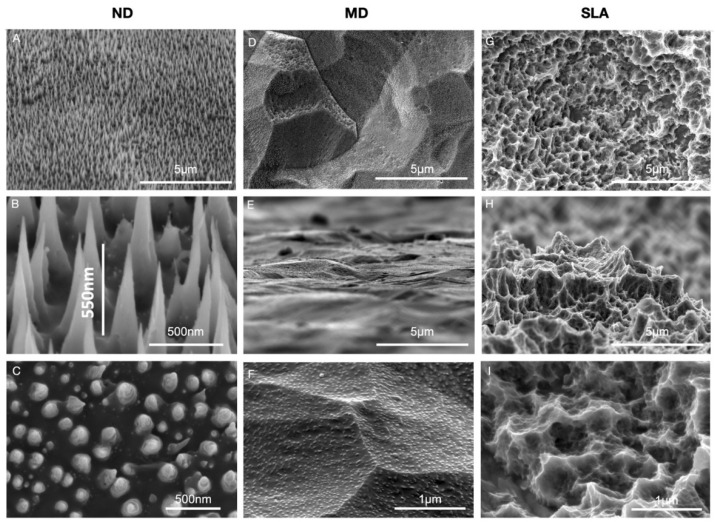
Top-view SEM images of nanostructures of up to 500 nm in height—ND (**A**–**C**), machined titanium surfaces—MD (**D**–**F**), and SLA surfaces (**G**–**I**). Images (**A**,**B**) show views at a 52° tilt angle; (**E**,**H**) show views at an 85° tilt angle.

Figure 1 shows top-view SEM images of nanostructures of 500 nm in height (Figure 1A–C), machined discs (Figure 1D–F), and SLA surfaces (Figure 1G–I). Figure 1A,B show views at a 52° tilt angle, while Figure 1E,H show views at an 85° tilt angle. The height of the spikes was directly proportional to the exposure time, although the base size (~200 nm) was not influenced. As seen in Figure 1 (Figure 1A,B, and more precisely in Figure 1C), the spike density was not modified for 500 nm nanospikes, and was influenced by the exposure time. The height of 500 nm was achieved after 24 h exposure time. As seen in Figure 1D–F, MD had a polished and smooth surface with microgrooves, which were a result of the polishing process. In contrast, SLA discs (Figure 1G–I), which were produced by coarse grit-blasting with 0.25–0.5 mm corundum grit at 5 bar, followed by acid etching, had a high number of peaks/valleys across the surface.

#### 4.1.2. AFM

**Figure 2:**
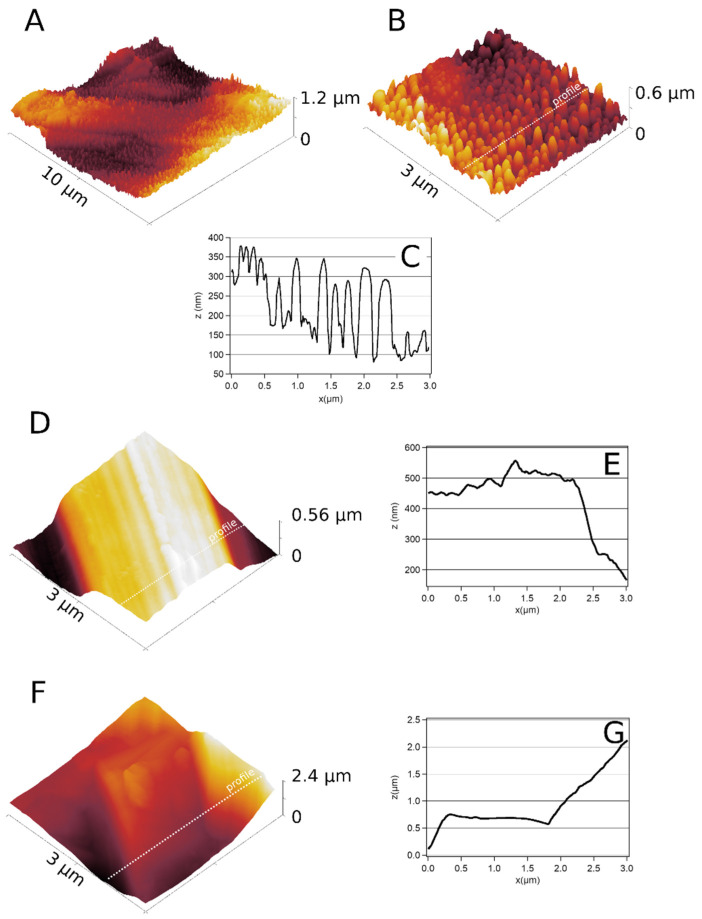
Intermittent-contact AFM topographic images taken with a sharp, high-aspect-ratio AR5-NCLR cantilever from Nanosensors (**A**,**B**). In both images, spikes of up to 400 nm are visible on the nanostructured titanium surface. Panel (**C**) shows the topographic profile along the line marked in (**B**). In comparison, (**D**,**F**) show (3 µm × 3 µm) topographic images and corresponding topographic profiles ((**E**,**G**), respectively) of machined and SLA surfaces, respectively.

The topography of the spikes was also investigated using atomic force microscopy (AFM) in intermittent-contact mode (Figure 2). These characterizations were carried out on 500 nm tall spike samples. For scan frame sizes larger than 5 × 5 μm, the roughness was dominated by large-scale hills and valleys. For frame sizes of 5 × 5 μm and smaller, the average roughness was dominated by the height of the titanium spikes (Figure 2A,B). When the scanned area was reduced further, the average roughness leveled off at a value of ~200 nm, which is consistent with the SEM images (not shown here). Figure 2D,F show topographic images and profiles (Figure 2E,G) of MD and SLA titanium surfaces. The MD surface showed a trench-like characteristic for mechanical polishing, whereas chemical etching of the SLA surface led to the formation of a rough surface.

The average roughness measured by AFM for ND, MD, and SLA surfaces is summarized in Table 1 for three different surface areas. The highest roughness values were reported for the SLA titanium surface, whereas the machined titanium showed the lowest average roughness. A significant difference in roughness was found between the SLA and MD/ND surfaces. The change in roughness was also correlated with adhesion.

**Figure 3:**
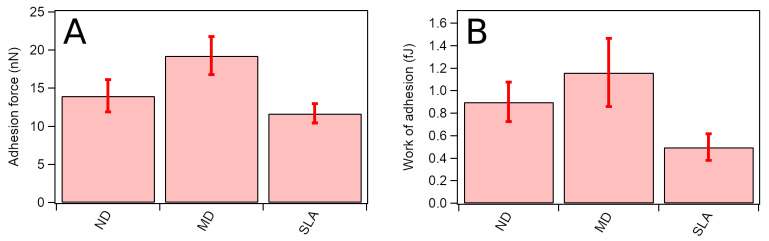
Adhesion forces (**A**) and adhesion work (**B**) measured between the silicon AFM tip and ND, MD, and SLA surfaces. The cantilever used was a PPP-Cont from Nanosensors with stiffness equal to k = 0.1 Nm^−1^. Thirty force—distance curves were measured on each sample in order to determine adhesion values.

By means of contact mode AFM, adhesion force and adhesion work measurements were performed between ND, MD, and SLA surfaces and the silicon AFM tip. The adhesion results are shown in Figure 3. The flat MD surface showed the largest adhesion, whereas on the rough SLA surface the adhesion force and adhesion work were reduced.

### 4.2. Adhesion of P. gingivalis on Titanium Surfaces

#### 4.2.1. Oral Pathogen Morphology

**Figure 4:**
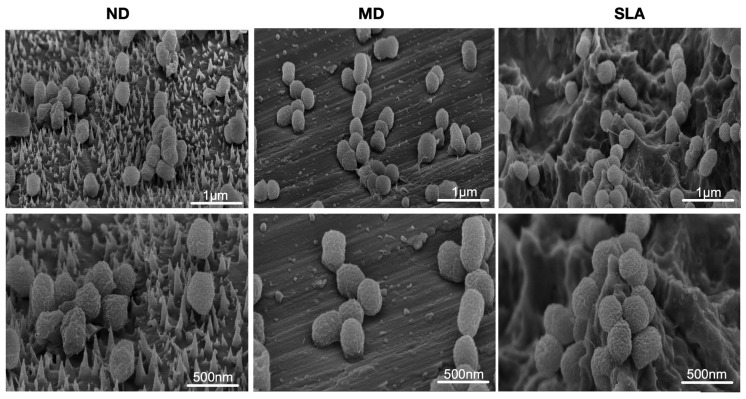
SEM scans of different titanium discs following incubation for 6 h with *P. gingivalis* cultures. The three top panels have bars indicating 1 μm for ND, MD, and SLA; the three panels at the bottom have bars indicating 500 nm for ND, MD, and SLA. All specimens were occupied by clusters of *P. gingivalis*. Additionally, *P. gingivalis* showed dysmorphism on ND.

SEM images revealed similar images between the control, MD, SLA, and ND surfaces. The heterogeneous surface observed here was confirmed by conventional culturing, showing high variance in bacterial adhesion and survival rates (Figure 4).

#### 4.2.2. Conventional Culturing Assays

The reduction in bacterial adhesion on ND in comparison to MD and SLA was 17 and 20%, respectively. No statistical differences in bacterial logarithmic count were found between groups (*p* > 0.05) following 6 h of bacterial adherence (Table 2).

### 4.3. Specimen Interaction with Human Gingival Fibroblasts (HGFs)

#### 4.3.1. Comparison of Specimens in MTT Assay

**Figure 5:**
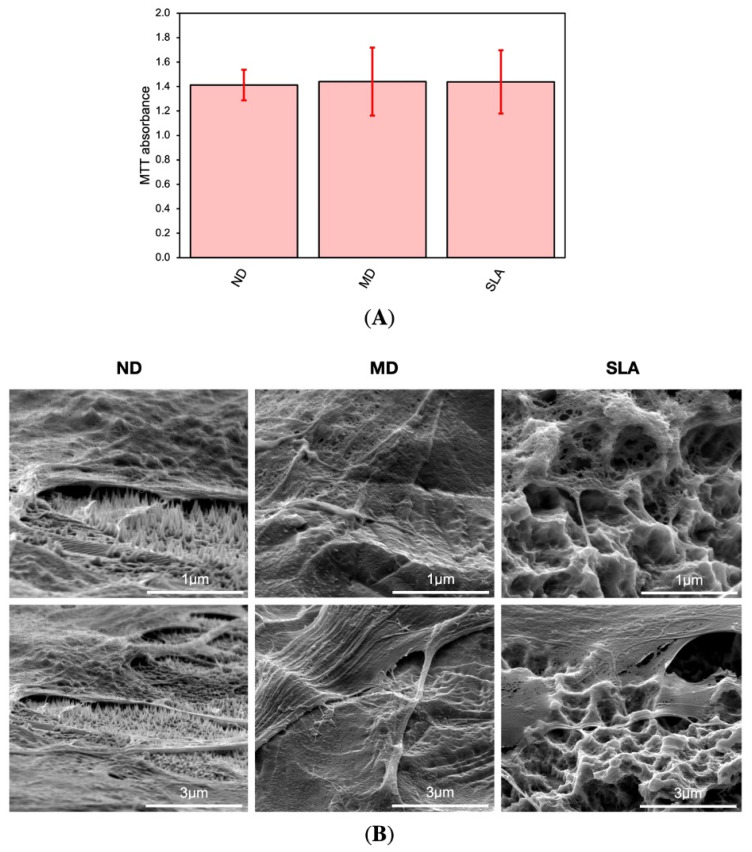
(**A**) Comparison of ND, MD, and SLA in the MTT assay. HGF cells were cultivated on discs for 72 h, and their viability was tested via MTT assay. Three independent experiments were performed in triplicate; *n* = 3, error bars represent SD. X-Axis: ND, MD control, SLA control. (**B**) SEM images of HGF-1 after 24 h on the tested materials: ND, MD, SLA. Abundant filopodia attachments were found on all samples. Portions of the HGF-1 cytoplasm exhibited a reticulated appearance. Through its sharp shape, the nanospike structure appears to perforate the fibroblast at some spots.

The incubation of HGF cells on ND, MD, and SLA surfaces did not influence their viability. Moreover, all discs showed very similar results, as absorbance peaks were almost identical between the three surfaces tested. Bar graphs representing the MTT assay results (Figure 5A) highlighted minimal discrepancies between the study and control groups, with such discrepancies not having any statistical significance.

#### 4.3.2. Cell Morphology

Cell morphology was qualitatively assessed via SEM after 24 h of culture. SEM images of ND, MD, and SLA showed that flattened and elongated HGF-1 cells adhered to the surfaces. Filopodia attachments were found on all samples; however, these attachments were more abundant on ND. MD showed spindle-shaped fibroblasts, whereas ND showed reticular-shaped HGFs. SEM images revealed that fibroblasts spread very flat and attach tightly to the smoother surfaces of MD and ND compared to SLA. However, HGF cells do not fully extend into the sandblasted and acid-etched morphology of SLA; moreover, filopodia attachments stretch over longer distances (Figure 5B).

## 5. Discussion

A new approach for creating a nanostructured surface with nanospike heights of 500 nm (ND) was successfully introduced in this study. Nanospikes can serve as a physicomechanical-based antibacterial measure against *P. gingivalis*, which plays a leading role in the development of peri-implantitis. Antimicrobial titanium surfaces can be developed via a spectrum of methodologies, e.g., glancing angle sputter deposition, nanoimprint lithography, hydrothermal manipulation, or helium ion bombardment, which was introduced in the present study. The ladder technique used to create such a nanospike surface proved to be successful, especially in terms of the ability to modify nanospike height as desired. The control group in this study consisted of smooth-machined titanium discs (MD) with a polished surface—corresponding to the machined transgingival part of an implant, typically utilized for tissue-level dental implants—and SLA titanium discs (SLA), which were sandblasted and acid-etched (Institute Straumann AG, Basel, Switzerland), corresponding to the osseointegrated part of a dental implant.

When compared to an unstructured machined titanium surface, the nanospike surface had no detrimental or cytotoxic effects on fibroblast cell viability. This characteristic is essential in order to allow proper implant establishment with the surrounding tissues, consequently increasing the chances of successful soft tissue integration [30]. Additionally, HGF-1 cells exhibited a spindle-shaped morphology, and were oriented along the grooves on MD, according to our findings. On the other hand, ND and SLA mostly showed cells with a reticular shape and the formation of filopodia, which is consistent with other recent studies in the field [29,31]. This might be explained by the applied helium ion bombardment, which may have partially removed the machining features on ND (as shown in Figure 1 and Figure 5B). These findings suggest that nanogrooves and machining features with the relevant morphological variations influence changes in cell alignment. Fibroblasts can respond to the micro- and nanotopography of the substratum surface, which is also known as ‘contact guidance’, referring to the orientation, changes in cell shape, polarity, and alignment of the cell as a result of the micro- or nanostructure of distinct surfaces [32]. Cells on smoother surfaces expand and establish a strong actin cytoskeleton to mechanically anchor themselves onto the topography, whereas topographic characteristics on rough surfaces are utilized to stabilize the cells [33,34].

Helium ion bombardment created a slightly higher surface roughness compared to the polished surface of MD, whereas SLA was 4.8× rougher. Several studies have found that the viability and proliferation rate of human gingival fibroblasts are significantly increased on polished surfaces when compared to alternative groups [35,36]. The cell response on various surfaces may not be ascribed to nanoscale roughness of the surface alone; rather, it appears that the variation in microprofile and chemical characteristics can alternate cell responses. It has been reported that an implant with a rough surface may exhibit a more prominent development of peri-implantitis than an implant with a smooth surface, although rough surfaces allow enhanced osseointegration [37,38]. In this regard, ND surfaces might be more favorable for peri-implant tissue health than implants with rough surfaces. Despite the rougher surface of ND in comparison to MD, no difference in the viability of HGFs could be detected, which is a promising sign for future application on the transgingival part of dental implants, as well as for possible soft tissue integration.

AFM adhesion force and work measurements were performed between ND, MD, SLA surfaces and the silicon AFM tip. The flat MD surface showed the greatest adhesion, whereas on the rough SLA surface the adhesion force and adhesion energy were reduced. This appears to be directly related to the surface roughness. Due to the flat shape of MD, the AFM tip seems to have a larger surface area to adhere to, despite the increased surface created by sandblasting and acid etching for SLA, or by helium ion bombardment for ND. Our findings suggest that adhesion force and energy are affected by both micro- and nanostructure. A recent study by Lagonegro et al. suggested that osteoblasts preferentially adhere to the peaks of the microstructure of SLA [39]. These findings are highly comparable to the data provided in this study, as SEM images revealed that the fibroblasts spread very flat and adhere tightly to the smoother surface of MD and ND compared to SLA. However, HGF cells do not fully extend into the sandblasted and acid-etched morphology of SLA, and filopodia attachments stretch across larger distances.

ND successfully induces dysmorphisms within *P. gingivalis* cultures following an incubation period of six hours. Bacteria attached to the ND surfaces appeared to be deflated or stretched when compared to bacteria incubated on the machined control surfaces, with the tips of the nanopillars protruding through the top sides of the collapsed cells; this outcome is consistent with earlier published studies [40,41]. Conventional culturing showed a bacterial reduction of 17 and 20% compared to MD and SLA, respectively. Our findings reveal a percentage-wise antibacterial tendency, but not a sufficient logarithmic reduction based on colony-forming units to claim sufficient antibacterial efficacy. Previous research investigated the effects of nanospike surfaces on *S. aureus* and *E. coli*, whereas the present work is centered on *P. gingivalis.* One probable cause is the difference in size and diameter between *E. coli* (2 m long, 0.25–1 m diameter) and *P. gingivalis* (1.5 m long, 1 m diameter), which may require a distinct nanostructured design of the nano–micro-hierarchical surface to boost antibacterial effectiveness. These distinct orders of magnitude might have a significant influence on the nanostructure’s properties against various pathogens [42]. Other bacterial traits, such as structural and morphological properties, membrane thickness, or elasticity, may either hinder bactericidal action or encourage initial attachment. The murein thickness of bacteria is critical to the efficacy of antibacterial nanostructures. Ivanova et al. (2012) demonstrated that cicadae wings exhibit bactericidal effects on *P. aeruginosa*, with a death rate of approximately 2.05 × 10^5^ CFU/(min × cm^2^), while the present study showed a death rate for *P. gingivalis* of approximately 1.3 × 10^8^ CFU/(min × cm^2^), which is three magnitudes higher [40]. This surface is made up of nanopillars with diameters ranging from 50 to 250 nm, with varying heights, spacing, and densities. In comparison to the nanospike surface in the present work, the nanostructures were nearly half the length, implying that they were more efficient in rupturing the murein wall of the mentioned pathogen (*P. aeruginosa*). This suggests that when designing a nanostructure to combat multiple pathogens, a one-size-fits-all approach is not feasible.

A recent study by Bright et al. concluded that bactericidal efficacy is reduced in a stepwise manner as bacterial concentrations are increased, with declines in bacterial viability observed for *S. aureus* at 10^5^ CFU/disc and *P. aeruginosa* above 10^6^ CFU/disc. Surprisingly, biofilm depth analysis revealed a decrease in bacterial viability. *P. gingivalis* showed a 1.3 × 10^8^ CFU adherence after 6 h in the present study, which is two orders of magnitude greater than in the work by Bright et al. The large number of colony-forming units at the start of the experiment could potentially be a reason for the different outcome observed when compared to other study findings, as suggested by Bright et al. [43].

The SEM scans, on the other hand, revealed that the ND study group had heterogeneity in nanospike growth across all examined regions for each disc. Such heterogeneity in nanospike surfaces could possibly be the main reason for the lack of the tangible antibacterial effect desired from such nanostructured biomaterials. Rather than providing efficient antibacterial properties, ND did lead to morphological deformities in *P. gingivalis*. It has been shown that stretching from the nanopatterned surface leads to deformation and mechanical rupture of *P. aeruginosa* [40,44]. During the adhesion process, if the degree of stretching is sufficient [45], it will lead to cell rupture and death; nevertheless, this was not the case for the nanostructures used in the present study, which could be explained by the different morphology of the pathogen used in this study. Despite such limitations, the results obtained from this study are highly comparable, and are equivalent to similar parallel studies on the development of antibacterial nanospike surfaces for dental implants. This study achieved nanospikes of up to 500 nm in length via a newly introduced technique, while in the recent study carried out by Elliott et al. such nanospike lengths peaked at 1250 nm [21]. In addition, although the investigation carried out by Elliott et al. did achieve success, with 11–40% of bacterial cultures being killed when attempting attachment on the nanospike surface [21], this reduction is highly comparable with the results (17–20%) presented in this study—although a percentage reduction is not enough to claim antibacterial properties; rather, a logarithmic bacterial reduction is a defining marker.

Following such additional knowledge, future research endeavors should focus on optimizing the nanospike deposition methodology in order to enhance nanospike surface homogeneity across the target regions. Other optimizations could include modification of the inter-nanospike spacing as a means for further potentiating antibacterial effects against *P. gingivalis* and other pathogens. In addition, future assays focusing on biofilm exposure to nanostructured surfaces should include various incubation time periods to increase bacterial load in order to simulate the accumulation of biofilm over time. This measure ensures possible acclimatization and establishment/attachment of such bacterial cultures on the investigated biomaterials.

## 6. Conclusions

In essence, the nature-inspired technology of nanostructured surfaces for the purpose of providing antibacterial properties against microbial threats is still in its infancy; 500 nm nanospikes have a tendency to hinder *P. gingivalis* adhesion, whilst also not interfering with the viability of gingival fibroblasts. However, nanospike length and spacing must be addressed in future studies in order to further influence the level of *P. gingivalis* adhesion reduction and the strength of HGF-1 adhesion.

The results demonstrated in this study confirm steady progress in the beneficial potential and clinical value of implementing such novel biomaterials for use in dental implants and other medical aids that can provide an increased quality of life within recipient patients in the near future.

## Figures and Tables

**Table 1:** The comparison of average roughness of ND, MD, and SLA surfaces measured via intermittent-contact AFM. The roughness depends on the scan area. For ND titanium surfaces and for large scan frame sizes, the roughness was determined by large-scale hills and valleys, whereas roughness for smaller scan areas was mainly due to the height of the titanium spikes.

Sample	Scan Area—(20 × 20) µm^2^	Scan Area—(10 × 10) µm^2^	Scan Area—(3 × 3) µm^2^
MD	0.41 µm	0.29 µm	0.27 µm
ND	0.98 µm	0.47 µm	0.23 µm
SLA	4.75 µm	1.65 µm	1.1 µm

**Table 2:** Table representing mean CFU/disc following *P. gingivalis* exposure for possible bacterial adhesion on ND, MD, and SLA, for five replicates. ND showed a reduction of 17% and 20% compared to MD and SLA, respectively.

Method	ND	MD	SLA
Conventional culturing (CFU/mL)	1.2 ± 0.7 × 10^8^	1.6 ± 0.5 × 10^8^	1.5 ± 0.7 × 10^8^

## Data Availability

Data is contained within the article or Supplementary Materials.

## Supplementary Materials

The following supporting information can be downloaded at: https://www.mdpi.com/article/10.3390/nano12071065/s1, Figure S1: Heated titanium samples were exposed to helium (He) plasma using an unbalanced magnetron sputtering source by controlling the temperature (350 °C), the negative voltage on the sample (−120 V) and the time of exposure, the surface developed a nanostructured pattern (data in submission process). Top view images for 24 h exposure (A–C) and for 48 h exposure (D–F). Images A, B, D and E are views at 52° tilt angle. The height of the spikes was directly proportional to the exposure time, though the base size (~200 nm) was not influenced. As seen in Figure 1 (A and B and more precisely in images C and F), spikes density was not modified for both heights and is influenced by the ex-posure time. Roughly, twice the number of spikes is visible for the 500 nm height in comparison to 1000 nm spikes.
