# Supplementary material for: Novel Titanium Nanospike Structure Using Low-Energy Helium Ion Bombardment for the Transgingival Part of a Dental Implant
Source: Nanomaterials (Basel). 2022 Mar 24;12(7):1065. doi: 10.3390/nano12071065 (PMC9000873; doi:10.3390/nano12071065)
Supplement: Supplementary file 1 [file nanomaterials-12-01065-s001.zip › nanomaterials-1651905-supplementary.pdf]

Supplementary Materials

Novel Titanium Nanospike Structure Using Low-Energy Helium Ion Bombardment for the Transgingival Part of a Dental Implant

Khaled Mukaddam [1,*], Monika Astasov-Frauenhoffer [2], Elizaveta Fasler-Kan [3,4], Laurent Marot [5], Marcin Kisiel [5], Roland Steiner [5], Fabien Sanchez [5], Ernst Meyer [5], Joachim Köser [6], Michael M. Bornstein [7] and Sebastian Kühl [1]

1 Department of Oral Surgery, University Center for Dental Medicine Basel (UZB), University of Basel, Mattenstrasse 40, 4058 Basel, Switzerland; sebastian.kuehl@unibas.ch
2 Department Research, University Center for Dental Medicine Basel (UZB), University of Basel, Mattenstrasse 40, 4058 Basel, Switzerland; m.astasov-frauenhoffer@unibas.ch
3 Department of Biomedicine, University Hospital Basel, University of Basel, Hebelstrasse 20, 4031 Basel, Switzerland; e.fasler@unibas.ch
4 Department of Pediatric Surgery, Children's Hospital, Inselspital Bern, University of Bern and Department of Biomedical Research, University of Bern, Freiburgstrasse 15, 3010 Bern, Switzerland
5 Department of Physics, University of Basel, Klingelbergstraße 82, 4056 Basel, Switzerland; laurent.marot@unibas.ch (L.M.); marcin.kisiel@unibas.ch (M.K.); roland.steiner@unibas.ch (R.S.); fabien.sanchez@unibas.ch (F.S.); ernst.meyer@unibas.ch (E.M.)
6 Institut für Chemie und Bioanalytik, Hochschule für Life Sciences, Hofackerstrasse 30, 4132 Muttenz, Switzerland; joachim.koeser@fhnw.ch
7 Department of Oral Health & Medicine, University Center for Dental Medicine Basel (UZB), University of Basel, Mattenstrasse 40, 4058 Basel, Switzerland; michael.bornstein@unibas.ch
* Correspondence: khaled.mukaddam@unibas.ch

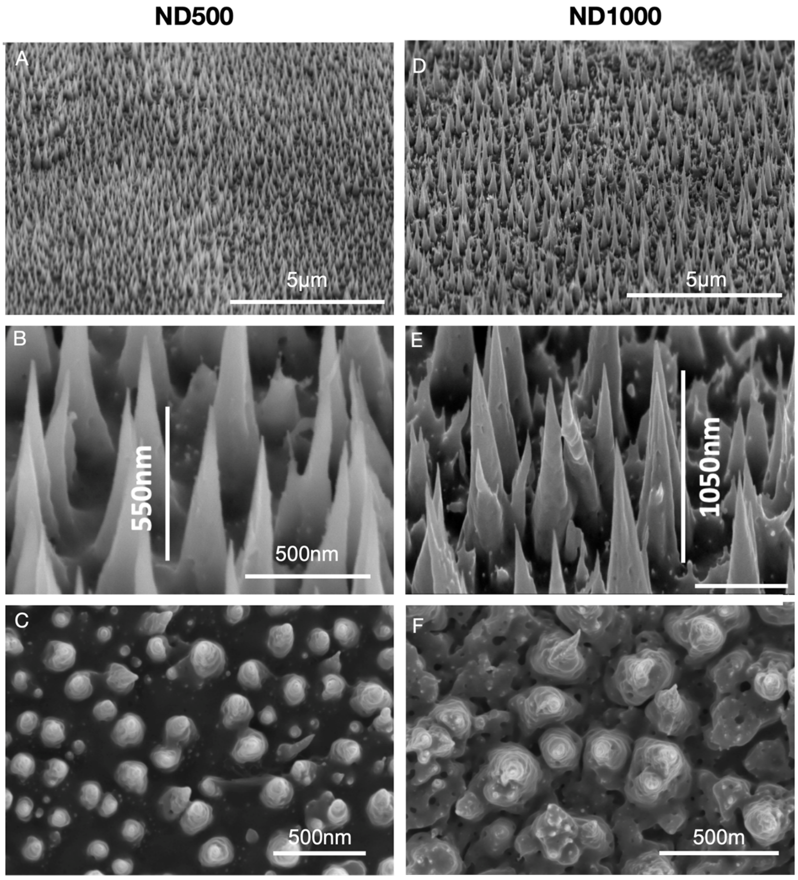

Figure S1. Heated titanium samples were exposed to helium (He) plasma using an unbalanced magnetron sputtering source by controlling the temperature (350 °C), the negative voltage on the sample (−120 V) and the time of exposure, the surface developed a nanostructured pattern (data in

submission process). Top view images for 24h exposure (**A**–**C**) and for 48 h exposure (**D**–**F**). Images A, B, D and E are views at 52° tilt angle. The height of the spikes was directly proportional to the exposure time, though the base size (~200nm) was not influenced. As seen in Figure 1 (A and B and more precisely in images C and F), spikes density was not modified for both heights and is influenced by the exposure time. Roughly, twice the number of spikes is visible for the 500 nm height in comparison to 1000 nm spikes.
